# Implementation and Extended Evaluation of the Euroimmun Anti-SARS-CoV-2 IgG Assay and Its Contribution to the United Kingdom’s COVID-19 Public Health Response

**DOI:** 10.1128/spectrum.02289-21

**Published:** 2022-02-23

**Authors:** Ashley David Otter, Abbie Bown, Silvia D’Arcangelo, Daniel Bailey, Amanda Semper, Jacqueline Hewson, Matthew Catton, Prem Perumal, Angela Sweed, Deborah Fox McKee, Jessica Jones, Heli Harvala, Abigail Lamikanra, Maria Zambon, Nick Andrews, Heather Whitaker, Ezra Linley, Alexander J. Mentzer, Donal Skelly, Julian C. Knight, Paul Klenerman, UKHSA Porton Euroimmun testing group, Gayatri Amirthalingam, Stephen Taylor, Cathy Rowe, Richard Vipond, Tim Brooks

**Affiliations:** a Diagnostic Support, Science Group, UK Health Security Agency, Porton, Porton Down, United Kingdom; b SARS-CoV-2 Serosurveillance Laboratory, UK Health Security Agency, Porton, Porton Down, United Kingdom; c Rare and Imported Pathogens Laboratory, UK Health Security Agency, Porton, Porton Down, United Kingdom; d NHS Blood and Transfusion, Microbiology Services, Colindale, United Kingdom; e UK Health Security Agency, Colindale, United Kingdom; f Seroepidemiology Unit, UK Health Security Agency, Manchester, United Kingdom; g Wellcome Centre for Human Genetics, University of Oxford, Oxford, United Kingdom; h Oxford University Hospitals NHS Foundation Trust, Oxford, United Kingdom; i Peter Medawar Building for Pathogen Research, Nuffield Department of Medicine, University of Oxford, Oxford, United Kingdom; j Nuffield Department of Clinical Neurosciences, University of Oxford, Oxford, United Kingdom; k UK Health Security Agency (UKHSA), Porton, Porton Down, United Kingdom; l Pathogen Immunology, UK Health Security Agency (UKHSA), Porton, Porton Down, United Kingdom; Regional Centre for Biotechnology

**Keywords:** SARS-CoV-2, assay development, coronavirus, immunoassays, neutralizing antibodies

## Abstract

In March 2020, the Rare and Imported Pathogens Laboratory at the UK Health Security Agency (UKHSA) (formerly Public Health England [PHE]) Porton Down, was tasked by the Department of Health and Social Care with setting up a national surveillance laboratory facility to study SARS-CoV-2 antibody responses and population-level sero-surveillance in response to the growing SARS-CoV-2 outbreak. In the following 12 months, the laboratory tested more than 160,000 samples, facilitating a wide range of research and informing UKHSA, DHSC, and UK government policy. Here we describe the implementation and use of the Euroimmun anti-SARS-CoV-2 IgG assay and provide an extended evaluation of its performance. We present a markedly improved overall sensitivity of 91.39% (≥14 days 92.74%, ≥21 days 93.59%) compared to our small-scale early study, and a specificity of 98.56%. In addition, we detail extended characteristics of the Euroimmun assay: intra- and interassay precision, correlation to neutralization, and assay linearity.

**IMPORTANCE** Serology assays have been useful in determining those with previous SARS-CoV-2 infection in a wide range of research and serosurveillance projects. However, assays vary in their sensitivity at detecting SARS-CoV-2 antibodies. Here, we detail an extended evaluation and characterization of the Euroimmun anti-SARS-CoV-2 IgG assay, one that has been widely used within the United Kingdom on over 160,000 samples to date.

## INTRODUCTION

Since its emergence in Wuhan, Hubei province, China, in late 2019, the novel severe acute respiratory syndrome coronavirus-2 (SARS-CoV-2) has caused a global pandemic of coronavirus 19 disease (COVID-19). A sobering milestone of three million COVID-19-associated deaths globally was reached on April 16 2021, with a cumulative total of more than 150 million SARS-CoV-2 infections reported worldwide (1). The true scale of SARS-CoV-2 infections globally is likely to be much higher, with a substantial number of cases of mild or asymptomatic disease being undetected.

The United Kingdom’s first confirmed case of COVID-19 occurred in a person who had travelled from Hubei province to England on January 23, 2020 and became symptomatic 3 days later (2). By the end of February, all four countries of the United Kingdom had recorded COVID-19 cases and community transmission was established (3). Case numbers increased significantly in early March 2020, reaching a total of 6,650 laboratory-confirmed cases on March 23 (4), when a national lockdown was implemented. At that time, epidemiological modeling estimated that for every hospitalized COVID-19 case in the United Kingdom, there were a further 120–124 infected individuals undetected within the wider community (4, 5).

Individual laboratories began to investigate the use of in-house tests, and it became apparent that the United Kingdom would benefit from a nationwide service to identify the prevalence of SARS-CoV-2 cases, including asymptomatic ones. Identification of these cases would be essential to inform government policy decisions; to further scientific understanding about immune response evolution, longevity, and correlation to protection; and to estimate the true extent of the United Kingdom population’s exposure to SARS-CoV-2. Additionally, such a service could address related questions such as the performance of alternative SARS-CoV-2 serology assays. However, this work was complicated by logistical issues including the availability of relevant test kits, which were in global demand, and the availability of relevant sample sets to validate new assays.

Described here is the extended evaluation of the SARS-CoV-2 Euroimmun IgG assay, in addition to establishment of a sero-surveillance laboratory using this assay for over 12 months.

## INTRODUCTION OF AN ANTI-SARS-COV-2 ASSAY

The Rare and Imported Pathogens Laboratory (RIPL) at UKHSA Porton Down was tasked by the UK Government’s Department for Health and Social Care (DHSC) with providing a SARS-CoV-2 serology service in support of a national serosurveillance program. Testing on this scale would require the use of commercial CE-marked serology kits to guarantee the volumes of supply required, the possibility for automation, and the necessary quality control of production batches, with our initial focus on an IgG specific assay. An initial market assessment identified only three companies with production kits available. Of these three kits, one targeted antibodies (IgG) against the SARS-CoV-2 spike protein S1 domain, one targeted nucleocapsid, and the third detected antibodies to an unspecified SARS-CoV-2 antigen. While there is merit in having assays that target different antigens for the determination of differential antibody responses to SARS-CoV-2 infection, the use of spike was judged to be critical for virus entry to cells, and therefore an assay detecting spike protein antibodies seemed the most attractive for initial use. Thus, the Euroimmun IgG assay (targeting the S1 domain) was selected for the urgent operational requirement and to also provide an indication of the immune profile of subjects.

The laboratory had available existing Stratec Biomedical Gemini platforms (Launch Diagnostics, UK) capable of performing automated ELISAs. After initial ELISA optimization on the Gemini platform, the first samples were tested to generate initial verification and evaluation data, using samples obtained from patients early in infection and with limited or incomplete clinical data through collaborators/hospitals.

On March 30, 2020, UKHSA Porton Down went live with a COVID-19 serology service for the United Kingdom, with an initial capacity of ∼800 samples a day. This was later expanded upon, with the addition of two further Gemini platforms, doubling capacity to ∼1,600 samples a day. Just over 12 months later, as of April 31, 2021, over 160,000 samples have been analyzed through this assay and workflow, providing answers to key questions about disease spread and the seroprevalence of SARS-CoV-2 infection, as well as informing outbreak management activities. To date, the Euroimmun assay has helped to deliver several significant studies for the UK population, covering population serosurveillance (5); health care worker exposure (6); selection of donors for convalescent plasma (7–10); characterization of reference standards; and research to determine the durability of the immune response (6, 11, 12).

## EXTENDED VERIFICATION OF THE EUROIMMUN ASSAY

**FIG 1:**
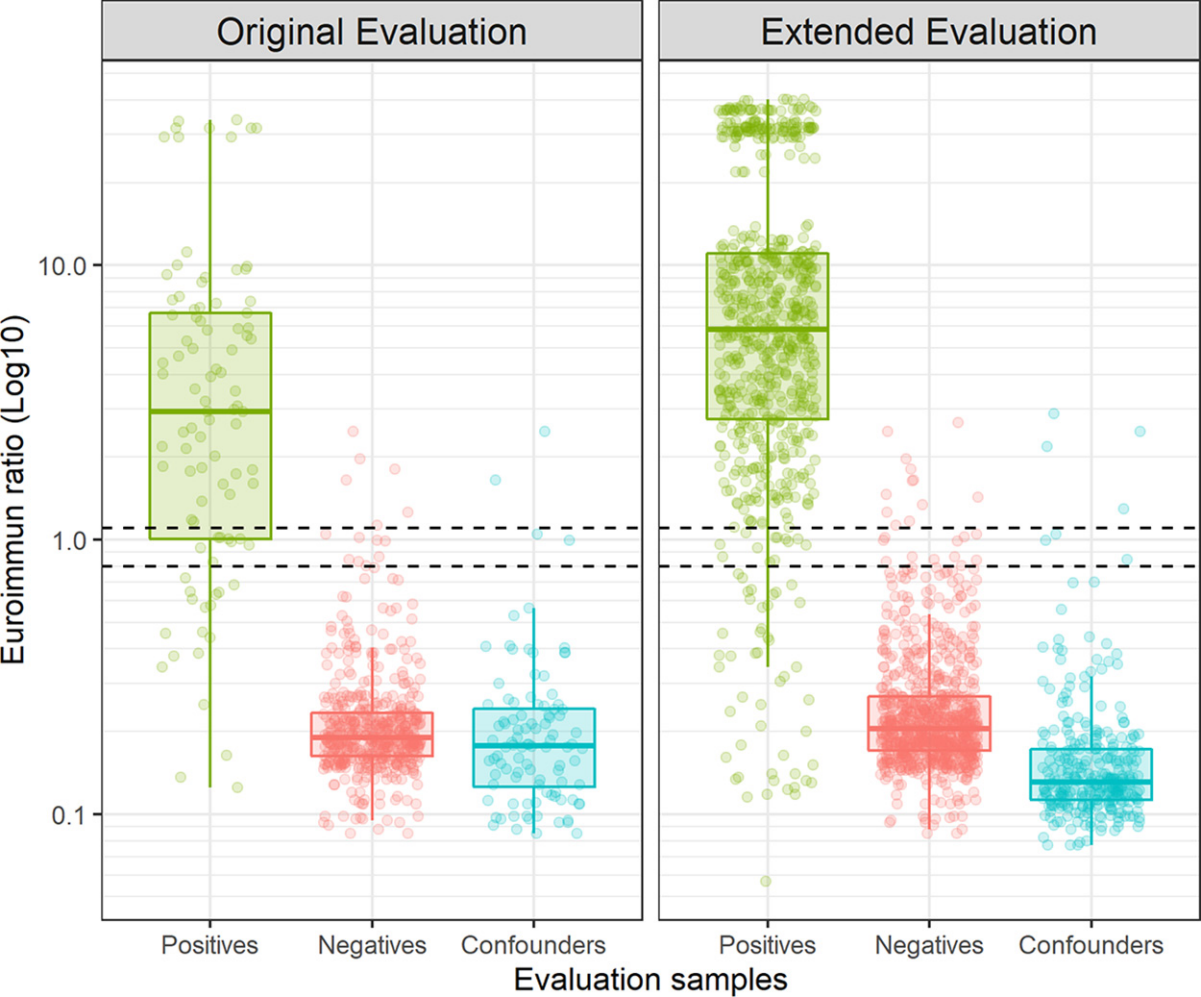
Distribution of evaluation sample results split according to (left) original evaluation (*n* = 692), and (right) extended evaluation (*n* = 1,799). Dashed lines represent the indeterminate range of the Euroimmun assay (ratio or index or s/co ≥0.8 and <1.1).

In our original small-scale evaluation of the Euroimmun assay, reported in June 2020 (13) and derived from samples obtained early in the outbreak, 100 serum samples from SARS-CoV-2 PCR-positives patients (where the time between sampling and symptom onset was known) were used to determine assay sensitivity, while 399 prepandemic negative serum samples, 50 serum samples from 2015 seronegative for Borrelia burgdorferi and 50 confounder samples (with previous confirmed VZV, EBV, CMV infection or rheumatoid positive) were used to determine specificity (left panel on Fig. 1). Of the SARS-CoV-2 positive panel, 100 serum samples were initially used, but this was later reduced to 93, as 7 were found to not have a PCR-confirmed prior SARS-CoV-2 infection. Of the positive samples, 67/93 tested positive (≥1.1 ratio) on the Euroimmun assay, giving an overall sensitivity of 72.0% (95%CI 61.78–80.86) (Table 1, Fig. 3, top panel). When analyzing only samples taken ≥14 days post-symptom onset, the sensitivity was 73.42% (95%CI 62.28–82.73), and at ≥21 days, the sensitivity was 74.67% (95%CI 63.30–84.01) (13).

**TABLE 1:** Sensitivity of the Euroimmun assay from the original UKHSA evaluation and the extended evaluation described here. Samples giving an indeterminate value are included with negative results

	All samples			Samples >14 days			Samples >21 days		
Panel	*n*	sensitivity (%)	95% CI	*n*	sensitivity (%)	95% CI	*n*	sensitivity (%)	95% CI
Original (16)	93	72.00	61.78% to 80.86%	83	73.42	62.28% to 82.73%	77	74.67	63.30% to 84.01%
Extended	685	91.39	89.03% to 93.38%	600	92.74	90.49% to 94.60%	579	93.59	91.41% to 95.36%

Subsequent to our initial establishment of the serosurveillance service using the Euroimmun assay, further commercial immunoassay platforms and kits were evaluated at the request of the DHSC to inform decisions on the use of high-throughput assays by NHS laboratories for the detection of anti-SARS-CoV-2 antibodies in patient samples (14, 15). As part of this work, we extended our evaluation of the Euroimmun assay. Due to the limited samples and volumes available for the evaluation of high-throughput serology platforms, the panels differed slightly between our initial Euroimmun evaluation and subsequent other commercial assay evaluations performed by UKHSA Porton Down (16). For the extended evaluation, an additional 592 serum samples were obtained from those with previous PCR-confirmed SARS-CoV-2 infection that were used for the different verifications of the higher-throughput platforms (including a head-to-head study [14]) were tested using the Euroimmun anti-SARS-CoV-2 IgG assay Fig. 1 (right panel).

Using this larger panel, the overall sensitivity of the Euroimmun assay was found to be 91.39% (95% CI 89.03% to 93.38%), increasing to 92.74% when using samples with an onset of ≥14 days (95% CI 90.49% to 94.60%) and 93.59% when using samples with an onset of ≥21 days (95% CI 91.41% to 95.36%), markedly improved from the original evaluation (Fig. 1 and 3, Table 1).

Additionally, the specificity panel was expanded from 499 to 1,114 serum samples: 303 confounders (from participants with previously confirmed CMV, VZV, EBV infection or Rheumatoid positive samples), 711 prepandemic negatives, and 100 Lyme disease negative samples from 2015. Using this panel, a specificity of 98.56% was calculated (95%CI 97.65–99.17%, *n* = 1,114), similar to that from the original evaluation (specificity of 99.0%; 95%CI, 97.5–99.7%).

**FIG 2:**
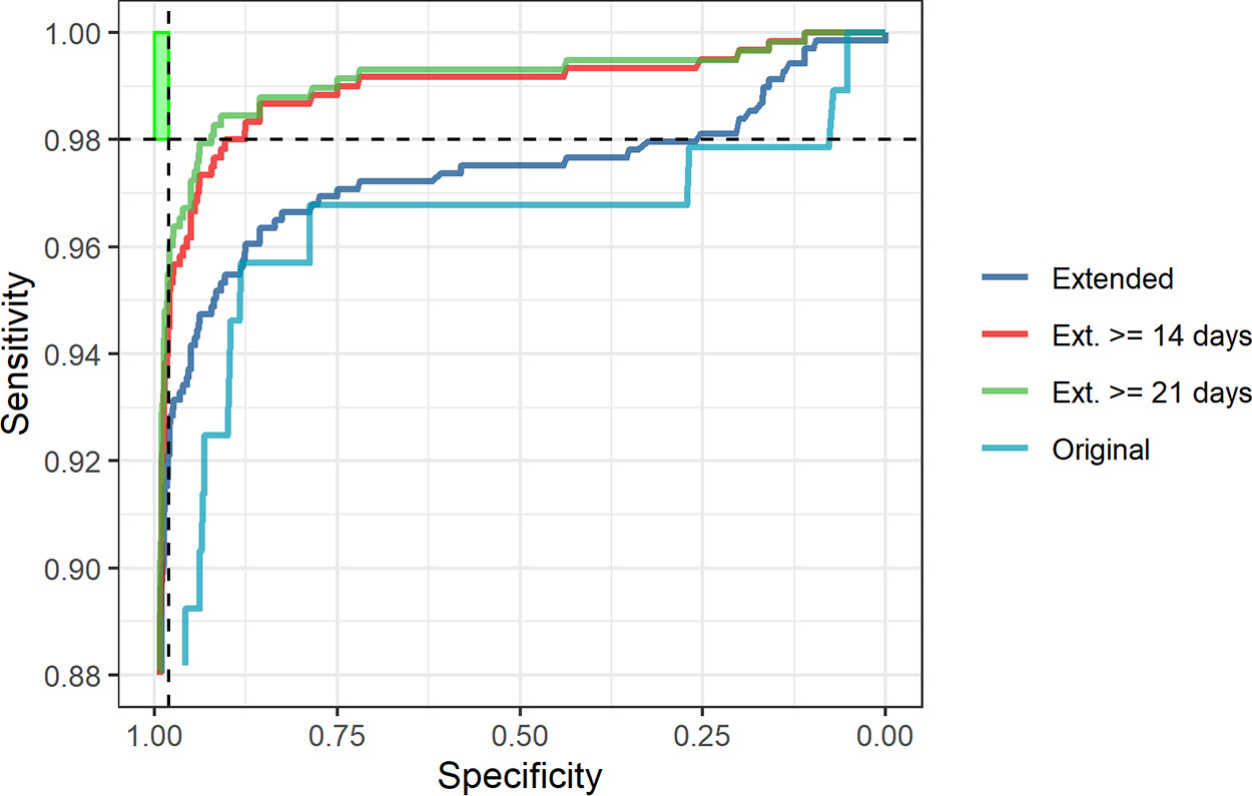
ROC curves for the Euroimmun assay. Curves are shown for the original (*n* = 592) and extended evaluations (*n* = 1,799), with the extended evaluation further split to show samples with intervals of ≥14 (*n* = 1,714) or ≥21 days (*n* = 1,693) after symptom onset. The green box indicates the MHRA Target Product Profile of serology assays (>98% sensitivity and specificity) at >20 days.

Receiver operating characteristic (ROC) analyses showed the highest area under curve (AUC) value was obtained (0.9902) when using samples from the extended evaluation panel with an interval of ≥21 days from onset of symptoms, while the original evaluation showed the lowest AUC (0.9584) (Table 2, Fig. 2).

**TABLE 2:** ROC analysis values for each of the evaluation panels, with the extended evaluation described here split by total and intervals of ≥14 and ≥21 days

Evaluation	Panel	Area	*P* value	95% CI	Negatives (incl. confounders)	Positives
Original (16)	Total	0.9584	<0.0001	0.9266 to 0.9902	499	93
Extended	Total	0.9729	<0.0001	0.9631 to 0.9827	1114	685
	≥14 days	0.9886	<0.0001	0.9826 to 0.9946	1114	600
	≥21 days	0.9902	<0.0001	0.9846 to 0.9958	1114	579

**FIG 3:**
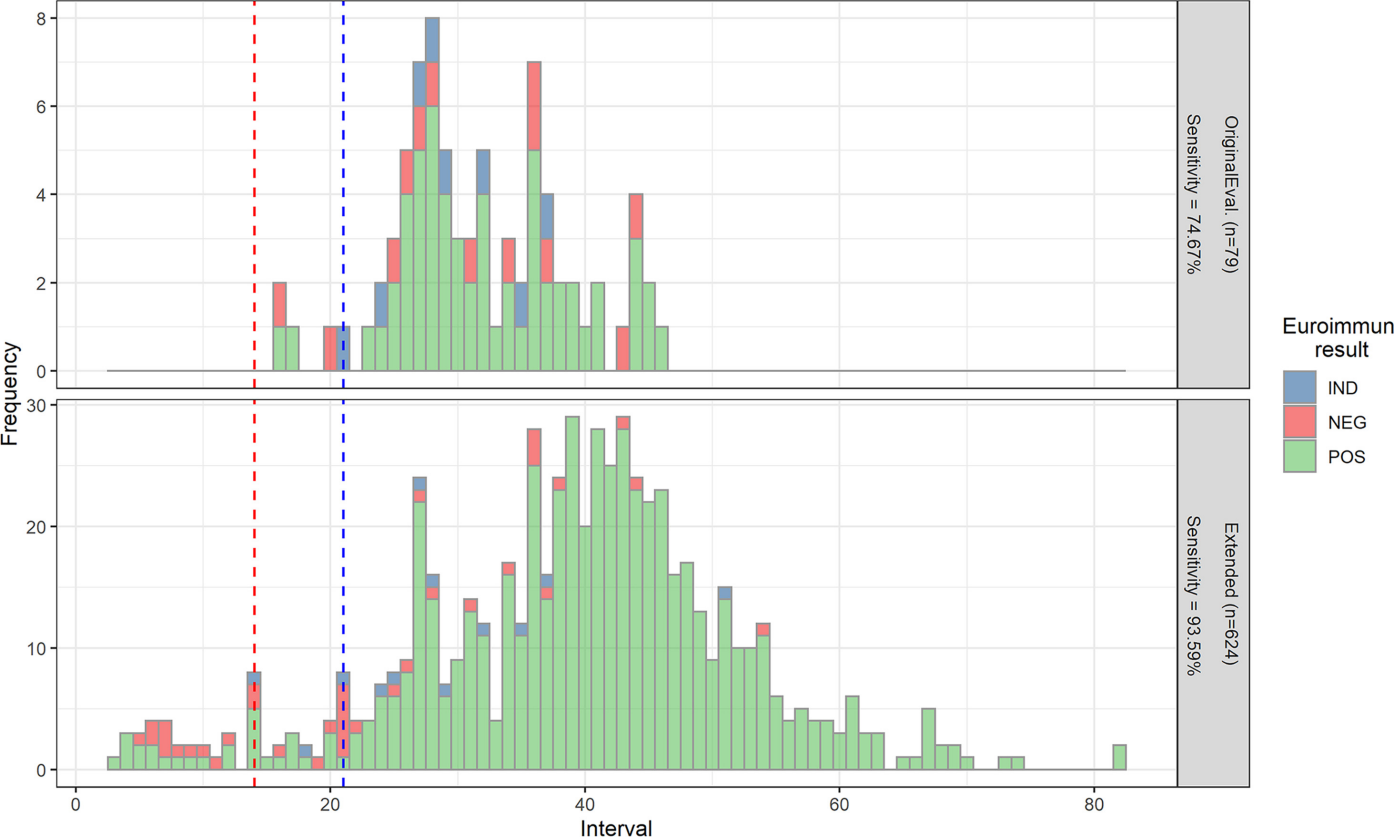
An overview of samples with days since PCR positive known plotted by interval distribution and evaluation panel, with coloring by the Euroimmun assay result. Top: Original evaluation (*n* = 79). Bottom: Extended evaluation (*n* = 624). The sensitivity at an interval of ≥21 days is included in each graph, in line with the MHRA TPP. Red dashed line indicates 14-day interval, blue dashed line indicates 21-day interval.

When examining the temporal distribution of the samples used for each of these evaluations, there was variation in the interval since disease/symptom onset (Fig. 3). Initial evaluation samples gave the highest frequency of reactive samples at 28 days, while the extended evaluation showed the highest interval frequency spanning 36–43 days after symptom onset. The majority of convalescent samples with intervals of <21 days tested positive (25 out of 53 samples).

This variation in interval distribution is reflected by the difference in the sensitivities seen between the two evaluations. Fig. 3 clearly shows that many of the initial UKHSA evaluation samples were limited in interval range, in part by sample availability, with samples typically taken between 14 to 35 days after symptom onset. This may account for differences in sensitivities, with some samples potentially taken too soon after onset of symptoms to identify any detectable antibody response. Conversely, many of the extended evaluation samples, by their nature, were taken from convalescent patients who had had sufficient interval post onset to generate a strong antibody response.

However, it should also be noted that no normalization for sample demographics (for example, differences in geographic location, gender, ethnicity, or patient age ranges) had been applied between data sets, so may have occurred and contributed to differences in sensitivity. Analysis of influence of demographic factors between sample sets is beyond the scope of this overview, but may be reported in more detail in individual studies. None of our evaluation sets included longitudinal samples from the same patient.

## FURTHER ASSAY CHARACTERISTICS

To determine the precision of the Euroimmun assay, five pooled patient samples were used in the absence of an international standard or calibrant at the time to generate a dilution series for linearity and to determine inter- and intra-assay variation, testing five replicates each over 5 days. The intra-assay and inter-assay variations were found to be <7% and <5% covariance (CV), respectively, indicating high precision when comparing samples across multiple days and replicate samples (Table S1 in the supplemental material).

**FIG 4:**
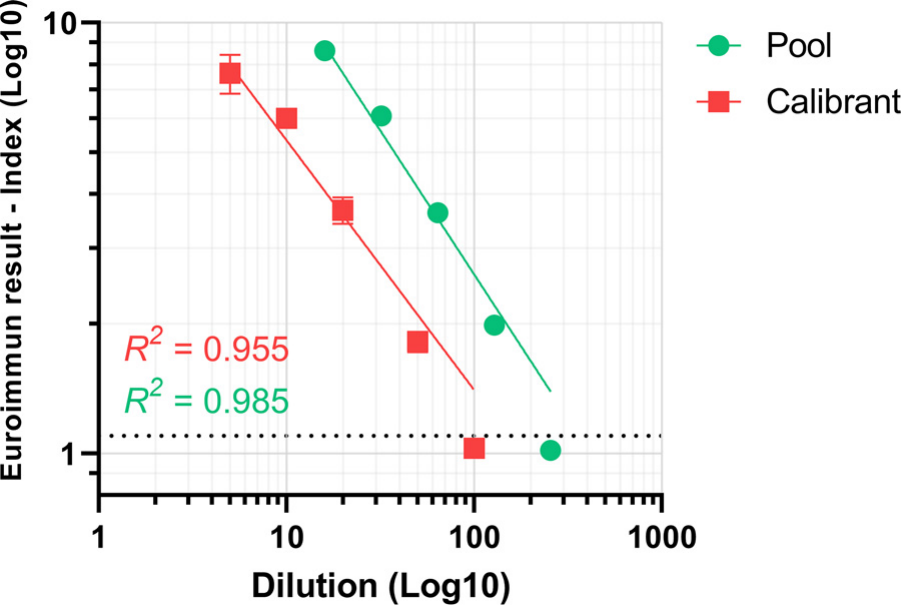
Dilutions and linear range of the Euroimmun assay, using pooled patient samples (pool; green) and the 20/162 NIBSC Anti-SARS-CoV-2 Antibody Diagnostic Calibrant (calibrant; red). Results >9 were removed due to saturated OD readings. Results with an index of ≥1.1 are considered positive while those <0.8 are considered negative, with the indeterminate range (0.8 to 1.1) shown with the dashed black line.

Similarly, the dilution series was used to determine the linear range of the assay, with an R^2^ value of 0.985 (Fig. 4). As more standards became available, the NIBSC Anti-SARS-CoV-2 Antibody Diagnostic Calibrant was serially diluted to generate additional linearity data, providing a similar R^2^ value of 0.955 (Fig. 4). From the pooled patient sera and the NIBSC calibrant dilutions, the linear range of the assay was determined to lie between index values of 1 and 9. It is noted that the reader configuration on the Gemini platform resulted in strong positive samples that saturated the optical density (OD) reader, and the software assigns an over-read OD value (e.g., 9.999), which can result in an index value of between 25 and 38 depending on the calibrator OD reading. The assay is routinely used in a qualitative mode (e.g., positive/negative interpretation), so saturating values were appropriately assigned as positive.

**FIG 5:**
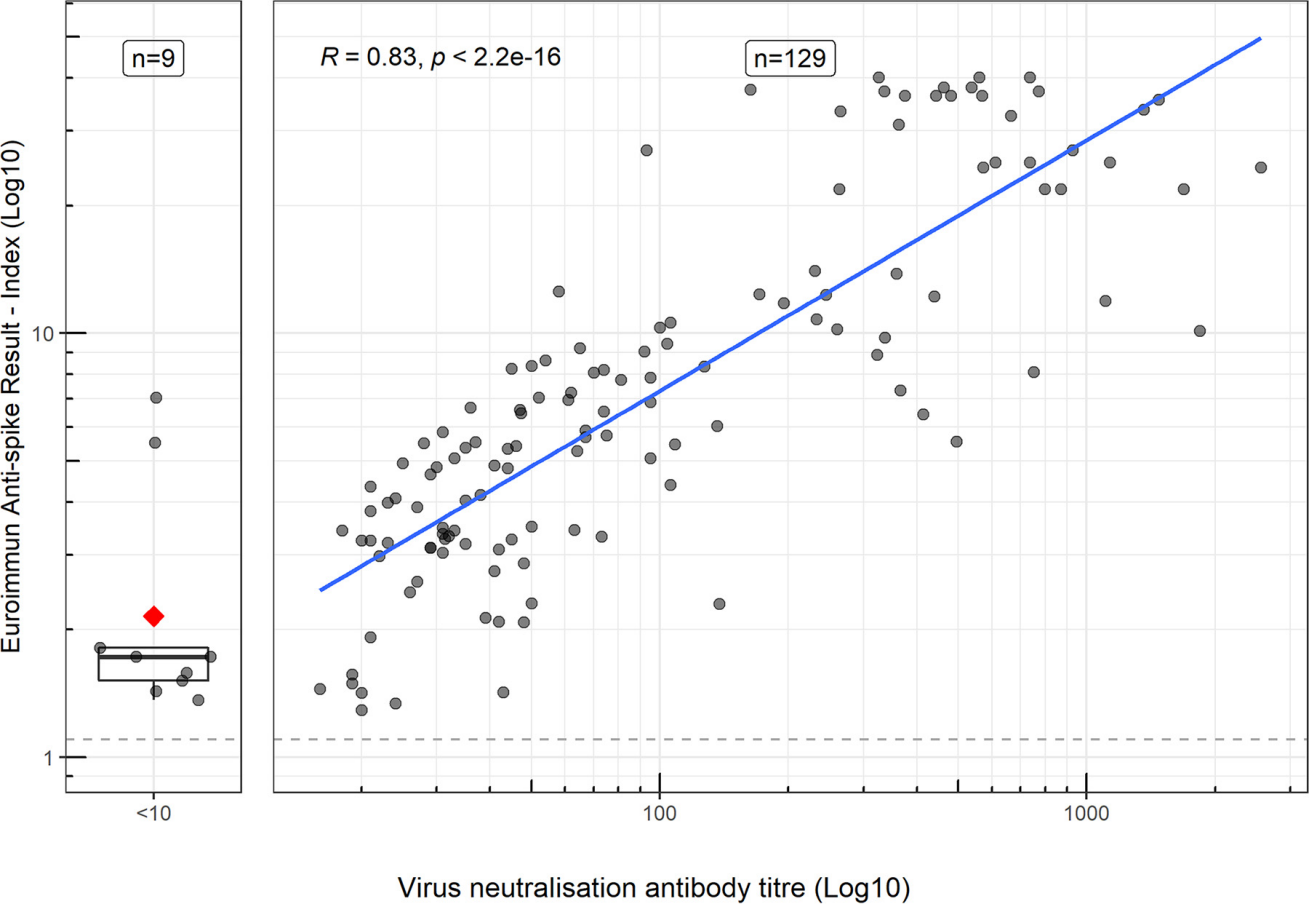
Comparison of convalescent blood donor serum samples tested using the Euroimmun anti-SARS-CoV-2 IgG assay (Log_10_ ratio value) and virus neutralization antibody titer (Log_10_). Good correlation (*R* = 0.83, R^2^ = 0.69) was observed between neutralization and the Euroimmun assay. Blue line indicates linear model, with 95% confidence interval (gray range) on the samples positive by neutralization and the Euroimmun SARS-CoV-2 IgG assay. Dashed line indicates the positive cut-off value (≥1.1 index), while the red square shows the mean of the samples negative by neutralization, but positive by Euroimmun assay.

Serology assays that have a strong correlation to neutralizing antibody titers are useful surrogates for live virus neutralization assays, which require significant containment level 3 work. Of the 138 samples that were tested by both neutralization and the Euroimmun assay, 129 were positive on both, while 9 were only positive by Euroimmun (Fig. 5), with seven of these samples giving an index result of 1–2 (close to the indeterminate range). There was a strong correlation between the Euroimmun results and the neutralization titer, with an R of 0.83 (R^2^ = 0.69).

## USE OF THE EUROIMMUN ASSAY IN THE UNITED KINGDOM

**FIG 6:**
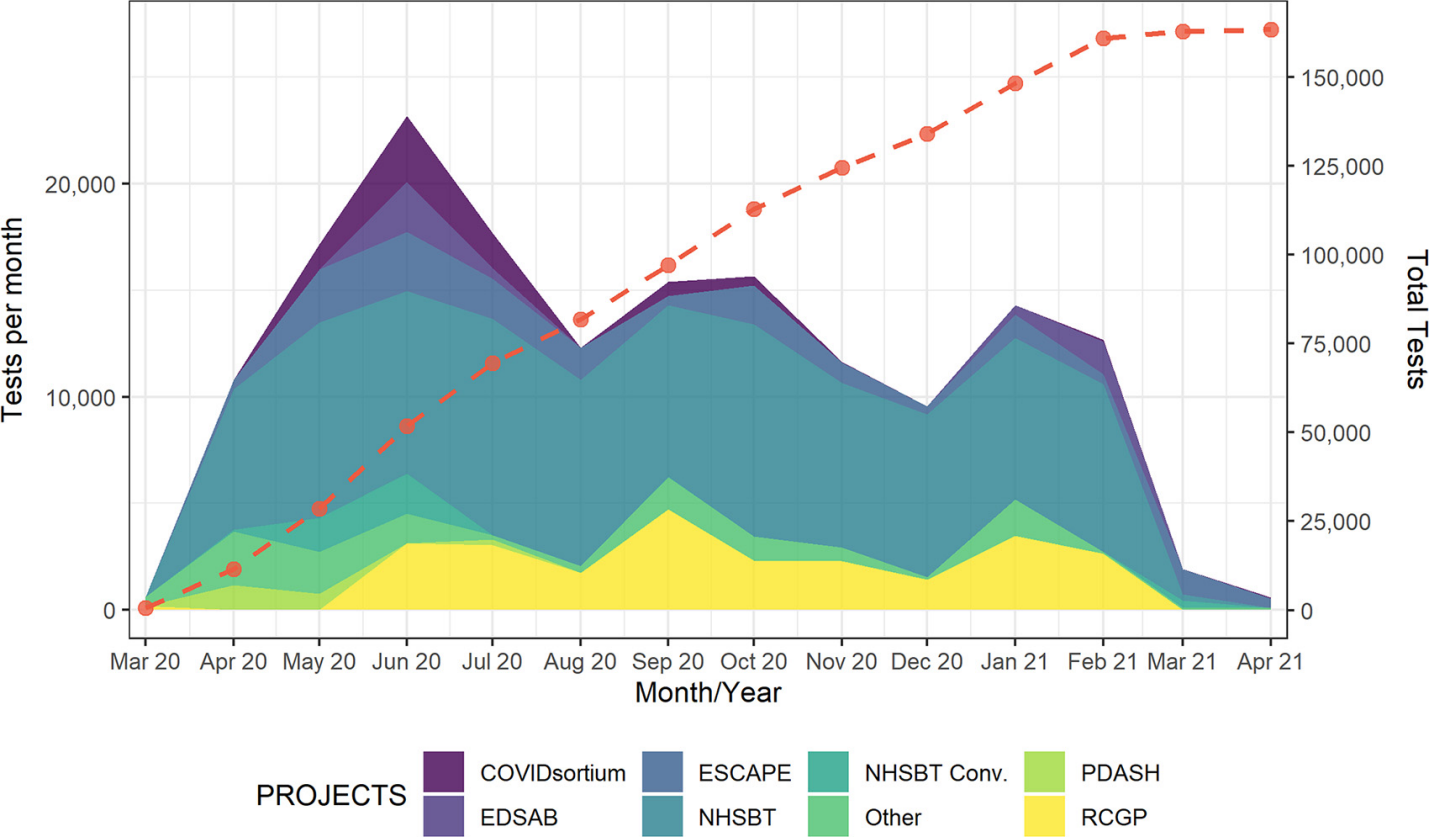
An overview of the number of samples tested using the Euroimmun assay at UKHSA Porton Down, with total tests (red-dashed line), spanning March 2020 to April 2021. Colors indicate different projects, as described in the text. Projects have since been moved to additional platforms as of March 2021.

A number of national and local projects with appropriate ethical approvals have been facilitated through this work, and Fig. 6 indicates the scope of Euroimmun testing performed by UKHSA Porton Down between March 2020 and April 2021. Descriptions of some of these projects are highlighted below, although more comprehensive analysis is published elsewhere from the respective project groups.

As part of a UK serosurveillance scheme, anonymized residual blood donor samples from people aged 17–84 from different regions of England were sourced from the NHS Blood and Transplant (NHSBT) service to provide additional venous blood samples to determine COVID-19 seroprevalence by age and region. This data began prior to other serosurveillance studies (for example, REACT, ONS) so provided UKHSA and external modeling groups with the only source of seroprevalence data for the United Kingdom at the time.

Approximately 10,000 samples were tested monthly, and positivity rate on the Euroimmun assay was determined on a weekly basis, to identify the population-level spread of COVID-19 and inform government decision making. Data were reported to the Public Health England seroepidemiology steering group (SSG) and scientific pandemic influenza group on modeling (SPI-M) and DHSC, as well as published in the weekly UKHSA (formerly PHE) COVID-19 report (5). There data helped to determine and understand the scale of asymptomatic spread of COVID-19 within the UK population, allowing UKHSA, DHSC, and modelers to assess age groups and areas that are driving transmission.

In addition to the NHSBT serosurveillance, blood samples were obtained from patients visiting primary care settings via the Royal College of General Practitioners (RCGP) Research and Surveillance Centre to determine COVID-19 seroprevalence, with a total of 24,927 samples tested since March 2020.

As part of a collaboration between UKHSA and NHSBT, convalescent plasma donor samples were tested to determine which donations should be provided for clinical trials assessing the therapeutic effects of convalescent plasma treatment of patients with COVID-19 (NHSBT Convalescent). The Euroimmun assay was chosen as the results showed a good correlation with neutralization assays (7, 8). Donations with a Euroimmun serology index result of >9.1 (later revised to 6.0) were shown to be associated with the presence of high neutralizing antibody titers of 1:100 or higher (7, 8) and were hence selected for trial use. Serological testing in support of this project began in April 2020, with the first patients receiving treatment at the beginning of May as part of the REMAP-CAP trial (10) and subsequently via the RECOVERY trial (9).

While laboratory antibody testing using ELISAs is considered the gold standard for determining population-level exposure to SARS-CoV-2, additional studies were conducted to determine the feasibility of using lateral flow device antibody tests. On request from DHSC, a study was performed on emergency service workers to evaluate the utility of home antibody testing kits: EDSAB-HOME (Evaluating Detection of SARS-CoV-2 AntiBodies at HOME). Capillary blood samples from >2,500 volunteers were tested on different lateral flow devices, and a contemporaneous venous sample was tested on the Euroimmun assay as the reference test (17).

From the start of the first COVID-19 lockdown in the United Kingdom (March 23, 2020), the COVIDsortium study (NCT04318314) collected serial samples from a cohort of 731 health care workers (HCW) with the aim of identifying host and pathogen correlates of protection and pathogenesis in mild/asymptomatic SARS-CoV-2 infection (18, 19). The Euroimmun assay was used throughout to characterize the serostatus of all study samples, which by completion of the final follow-up samples at 1 year, will number more than 13,000 samples. This data helped to define the proportion of this HCW cohort who seroconverted, and was used to monitor the longitudinal antibody response and rate of decline of anti-S1 IgG antibodies (6) and to investigate the relationship between peak anti-S1 IgG titer and neutralizing antibody titer (20).

In addition, a small project (ESCAPE) began in March 2020 as a mechanism to study antibody responses to infection. A cohort of Public Health England staff across 4 sites were recruited, with monthly blood donations taken as a way to understand the kinetics of antibodies post-infection (11).

A collaboration between UKHSA and Hampshire Hospitals NHS Foundation Trust (HHFT) provided 1,885 serial samples from 282 hospital in-patients with acute COVID-19 and 189 serial samples from 50 convalescent HCWs (the PDASH study). All participants had PCR-confirmed SARS-CoV-2 infections that ranged from asymptomatic to severe disease. The study used the Euroimmun assay to determine the time to seroconversion and to monitor the longitudinal antibody response in study participants.

Other projects included those used specifically for serology assay evaluations conducted by UKHSA Porton and UKHSA SEU, smaller research studies, and those conducted by hospitals and colleagues early in the pandemic.

## DISCUSSION

The Euroimmun assay has been fundamental to the United Kingdom’s public health response to COVID-19, with >160,000 samples tested from March 2020 to March 2021 (Fig. 6). This early availability of the assay was key to performing seroprevalence testing in the early stages of the pandemic, informing the policy and legislative responses of UKHSA, DHSC, and the UK Government. It also contributed to our understanding of immune responses during COVID-19 infection and recovery, as well as provided real-time (∼24 h) testing of convalescent plasma before being provided for use in clinical trials.

While many platforms and assays now exist for the detection of anti-SARS-CoV-2 antibodies (including those evaluated by ourselves [21]), there were limited choices available at the onset of the pandemic in the United Kingdom in March 2020. This highlights the need and basis for pandemic preparedness from an immunology and serology perspective, with the requirement for labs to be able to run high-throughput antibody tests on specimens from patients infected with newly emerging pathogens. Rapid immunology research on a new pathogen enables early understanding of potential immunity and informs early vaccine design. While in-house/research-based assays were available since January 2020, these require significant quality control to ensure consistent diagnosis across batches of samples and to ensure consistent assay sensitivity. Similarly, in-house assays can also suffer from logistical and supply issues for specially manufactured components (recombinant proteins, etc.). Commercial assays became rapidly available with the spread of SARS-CoV-2 in early 2020; however, supply and logistical issues were also evident with the global spread of SARS-CoV-2, so this should be a factor in deciding on assays for future serosurveillance if other pandemics occur.

In our original evaluation, we found an overall sensitivity of 72.0% (interval ≥14 days, 73.42%; interval ≥21 days, 74.67%) and a specificity of 99.0% for the Euroimmun assay, while the extended evaluation detailed here found an overall sensitivity of 91.39% (interval ≥14 days, 92.74%; interval ≥21 days, 93.59%) and a specificity of 98.56% (Table 1, Fig. 2). These data are supported by other studies, which have shown similar high sensitivities of 90% (22), 96% (23), and 100% (24). The initial evaluation suggested a moderate sensitivity for the Euroimmun assay, but this is likely a reflection of the early stages of the pandemic, when serum from patients with confirmed COVID-19 were extremely difficult to obtain, disease onset was poorly characterized, and little convalescent material was available. This limited the number of samples, as many of the original evaluation samples were obtained from patients with mild disease within the community, so in using these particular samples, this could have resulted in lower sensitivity than anticipated, due to mild infections and resulting in lower antibody responses than those with severe infections or those requiring hospitalization (25). In addition, timing between disease onset and sample date was not fully established for all patient samples, with many taken from patients too early since disease onset, whereby no robust IgG/immunological response is mounted.

Use of an extended sample panel has enabled us to better evaluate the Euroimmun assay. Although the Euroimmun assay showed improved sensitivity in our extended evaluation, in our analysis, it still does not meet the MHRA’s Target Product Profile (TPP) of >98% sensitivity recommendation for SARS-CoV-2 serology assays to determine if people have recent infection of SARS-CoV-2 (26); it does however meet the TPP of >98% specificity.

In addition to improved sensitivity using this extended panel, intra- and interassay variation was found to be low, with covariance of <7% and <5%, respectively. Using pooled patient samples and the NIBSC diagnostic calibrant, the linear range of the assay was also determined, enabling semi-quantitative comparisons (Fig. 4). Our results are consistent with other studies that show that only a defined range of the Euroimmun assay can be used to reliably infer a linear range (6).

As an antispike (S1 domain) antibody ELISA, the Euroimmun assay results also correlate with neutralizing antibody titers (*R* = 0.83, R^2^ = 0.69) as shown elsewhere (7, 8, 27), suggesting that this assay provides a useful surrogate for virus neutralization and possible correlation to immune protection (Fig. 5), without the requirement for containment level 3 facilities and live-virus culture. However, other pseudo-type assays have shown higher correlation (27), likely due to the different dynamics between antibody-binding assays and neutralization-based assays.

In addition, as an indirect sandwich ELISA, results are only semi-quantitative and display a maximum reading for optical densities due to reader saturation. In our equipment implementation, depending on the calibrator value, max OD values can then result in an index value of between approximately 26 and 38, preventing direct comparisons between samples unless the calibrator values are similar or are within the linear range of the assay, determined here to be between OD values of 1 and 9 (Fig. 4). If quantitative results are desired, our recommendation is to dilute samples that are strongly positive to within this range.

Notably, of the serology assays evaluated by UKHSA Porton Down to date (21), the Euroimmun assay format does not require the installation of dedicated machinery/specialist equipment, similar to other plate-based ELISAs. This enables wider use of the assay: it can be utilized in most laboratory settings using automated pipetting platforms (such as the Stratec Gemini), on the bench with manual pipetting, or within the field since only limited additional laboratory equipment such as a plate reader is required.

Serology assay selection for different requirements is complex (28). This initial program was principally focused on serosurveillance; further work is now under way at UKHSA Porton to determine antibody responses induced by vaccination (29), vaccine effectiveness (30), vaccine coverage, variant infection, and population spread (31), using a combination of serology assays targeting different antigens. With additional assays now available, we have expanded our assays to encompass those with improved sensitivity and specificity, with the capability to change rapidly, enabling better and more accurate analysis of the differing and refined responses to the SARS-CoV-2. Newer assays have enabled us to determine specific protein subdomain responses (e.g., anti-RBD), as well as using a combination of assays as a mechanism to differentiate between those with previous infection (presence of both antispike and antinucleocapsid antibodies), those with vaccination and no previous infection (presence of only antispike antibodies), and those with previous infection and vaccination (presence of both antispike and antinucleocapsid antibodies, with significantly higher antispike responses as reported elsewhere [29]).

## MATERIALS AND METHODS

### Serum samples.

Early samples were sourced from collaborating colleagues at UKHSA Porton Down, UKHSA Sero-Epidemiology Unit Manchester (SEU), and hospitals across England under Material Transfer Agreements (MTAs). These included residual diagnostic serum from SARS-CoV-2 PCR-confirmed samples from hospital in-patients (Guy’s and St Thomas’ Hospital, The Royal Free, Barnet Hospital and North Hampshire County Hospital, Basingstoke and John Radcliffe Hospital, Oxford), with data available on dates of collection versus onset of disease. All those with PCR-positive SARS-CoV-2 infection were interpreted as positive by their localized diagnostic laboratory.

Samples collected in Oxford were from individuals consented into 2 studies: Gastro-Intestinal Illness in Oxford: COVID substudy (Sheffield REC, reference: 16/YH/0247) and Sepsis Immunomics project (Oxford REC C, reference:19/SC/0296), as described (14). The large majority of samples were sourced from community cases of SARS-CoV-2 described within the FF100 (32) and the UKHSA (formerly PHE) serology assay evaluation supporting information (16). Of the Oxford patient cohort, 116 patients were classified as having mild disease, 15 severe, 13 asymptomatic, 6 critical, and 1 death.

To validate the Euroimmun assay (and future serology assays), prepandemic negative serum samples were also sourced to determine specificity, in addition to serum from individuals obtained prior to the SARS-CoV-2 pandemic but with prior infections such as CMV, VZV, EBV, or Rheumatoid positive. These were obtained from serum banks prior to the COVID-19 outbreak by the Royal College of General Practitioners (RCGP) for routine surveillance schemes and held by UKHSA SEU.

### Euroimmun anti-SARS-CoV-2 IgG assay.

All testing using the Euroimmun anti-SARS-CoV-2 IgG assay (EI 2606–9601 G, Euroimmun, Lübeck) was performed using an automated microplate processor (Stratec Gemini), which automates all aspect of the ELISA from sample dilution through to incubations, washing, reading, and analysis, according to the manufacturer’s instructions for use. Positive and negative kit controls provided by Euroimmun were included with every run, with commercial positive and negative anti-SARS-CoV-2 controls used in parallel (ACCURUN Anti-SARS-CoV-2 Controls Kit - Series 1000; SeraCare, Gaithersburg) for additional internal quality control. Control values were tracked using a Levy-Jennings plot with Westgard rules applied.

The assay is semiquantitative, with results expressed as recommended by the manufacturer as a ratio (index) of the extinction (optical density) of the patient sample (or control) over the extinction of the calibrator. Interpretation of samples was defined according to the manufacturer, with an index value of <0.8 defined as negative, >0.8 to 1.1 defined as indeterminate/borderline, and ≥1.1 interpreted as positive.

### Linearity and assay variation.

To determine the assay linearity, five high titer serum samples obtained through serosurveillance testing were pooled, with 2-fold dilutions generated, and each dilution was run in replicates of five. Similarly, when the NIBSC Anti-SARS-CoV-2 Antibody Diagnostic Calibrant (20/162, NIBSC, Potters Bar) became available, it was run in triplicate across 1:5, 1:10, 1:20, 1:50, and 1:100 dilutions. Results from both the pool and NIBSC calibrant were used to determine the linearity of the Euroimmun assay.

The pooled serum samples were additionally used to determine the intra- and interassay variation, with five replicates measured each day across 5 days.

### Microneutralization against SARS-CoV-2.

On a panel of 138 convalescent blood donors that were >28 days post-symptom onset, microneutralization titers against SARS-CoV-2 were determined as previously described (7), with neutralization titers compared against Euroimmun ratio results.

### Receiver operator curves (ROCs).

ROCs were generated in R (version 4.0.2) using the pROC package (33) (version 1.17.0.1).

### Ethical considerations.

Samples obtained through the NHSBT and RCGP serosurveillance schemes were approved by the UKHSA Research Ethics and Governance Group (REGG). Samples obtained from the University of Oxford were covered by ethical approval for individual projects registered under 16/YH/0247 and 19/SC/0296.
