# Supplementary material for: Implementation and Extended Evaluation of the Euroimmun Anti-SARS-CoV-2 IgG Assay and Its Contribution to the United Kingdom’s COVID-19 Public Health Response
Source: Microbiol Spectr. 2022 Feb 23;10(1):e02289-21. doi: 10.1128/spectrum.02289-21 (PMC8865481; doi:10.1128/spectrum.02289-21)
Supplement: SUPPLEMENTAL FILE 1 — Supplemental material. Download SPECTRUM02289-21_Supp_1_seq3.pdf, PDF file, 0.1 MB [file spectrum02289-21_supp_1_seq3.pdf]

Table S1: Precision results for the Euroimmun assay using five pooled patient sera. Samples were tested in replicates of five over five days. Intra-assay variation was <7%, whilst inter-assay variation was <5%

Sample		Day 1	Day 2	Day 3	Day 4	Day 5	Inter-assay Mean	Inter-assay SD	Inter-assay CV
1	Mean	9.00	8.43	8.59	8.43	8.64			
	SD	0.26	0.13	0.14	0.08	0.13	8.62	0.235	2.724
	CV	2.93	1.59	1.60	0.99	1.50			
2	Mean	6.28	5.95	5.99	6.04	6.12			
	SD	0.11	0.19	0.13	0.16	0.28	6.08	0.132	2.180
	CV	1.69	3.26	2.18	2.64	4.50			
3	Mean	3.72	3.52	3.55	3.67	3.67			
	SD	0.11	0.12	0.04	0.19	0.11	3.62	0.087	2.409
	CV	2.84	3.52	1.13	5.17	2.93			
4	Mean	2.07	1.90	1.99	1.99	1.99			
	SD	0.04	0.03	0.06	0.09	0.08	1.99	0.062	3.102
	CV	2.16	1.72	3.12	4.42	4.04			
5	Mean	1.07	0.98	1.02	0.99	1.03			
	SD	0.07	0.01	0.04	0.02	0.07	1.02	0.037	3.620
	CV	6.28	0.55	3.76	2.46	6.60			

Table S2: Determination of linear range of Euroimmun assay using serially diluted pooled patient sera. Pooled serum samples at dilutions of 2, 4 and 8 were removed due to OD saturation.

Source	Dilution	Index (mean)	SD
Patient pool	16	8.617	0.235
	32	6.077	0.132
	64	3.624	0.087
	128	1.987	0.062
	256	1.018	0.037
NIBSC calibrant	5	7.636	0.793
	10	6.000	0.279
	20	3.673	0.261
	50	1.820	0.100
	100	1.029	0.045
	200	0.614	0.013
